# Gut bioengineered models to study host-microbiota-probiotics interactions

**DOI:** 10.20517/mrr.2025.45

**Published:** 2025-10-22

**Authors:** Elise Delannoy, Alexandre Grassart, Catherine Daniel

**Affiliations:** Univ. Lille, CNRS, Inserm, CHU Lille, Institut Pasteur de Lille, U1019 – UMR 9017 - CIIL - Center for Infection and Immunity of Lille, F-59000 Lille, France.

**Keywords:** Probiotics, gut, host-microbiota interactions, gut-on-chip

## Abstract

The gastrointestinal tract is the major ecological niche in which gut microbes interact with epithelial and immune cells to maintain homeostasis in mammals. Moreover, probiotics modulate the gut microbiota and exert various health benefits after oral administration and persistence in the gut. Until now, animal models have been the gold standard for unravelling the mechanisms implicated in host-microbe interactions. However, their translational relevance to clinical trials and the associated ethical concerns underscore the need for alternative models. The emergence of microfluidic organ-on-chip technologies provides promising new alternative models to explore human host-microbe interactions while maintaining the tissue-level complexity and inter-individual variability. In this perspective, we discuss the potential of using mice, non-rodent models and gut-on-chip technologies to better characterize the interactions between the host, the gut microbiota, and orally administered probiotics, and to monitor microbial spatiotemporal dynamics at the tissue level.

## INTRODUCTION

The human gastrointestinal tract (GIT) includes different segments from the stomach to the colon and maintains homeostasis through interactions among epithelial cells, immune cells, and resident gut microbiota composed of bacteria, viruses, fungi, yeasts, and archaea^[1,2]^. Along its passage within the different intestinal compartments, the microbiota is exposed to variable parameters such as pH, flow rate, mucus layer, immune cells, digestive enzyme secretion, bile acids, and oxygen concentration, all playing a role in the composition and stability of the gut ecosystem^[3,4]^. In the small intestine, the secretion of digestive enzymes and bile, along with a short transit time, stimulates the renewal of bacteria^[5]^. Moreover, the immune cells of Peyer’s patches also contribute to homeostasis and microbiota diversity^[6]^. In contrast, the colon has the most stable microbial community with more than 400 species^[7]^. The Food and Agriculture Organization of the United Nations and the World Health Organization define probiotics as live microorganisms that, when applied in adequate amounts, provide health benefits to the host^[8-10]^. The most consumed probiotics in human nutrition belong to the genera *Lactobacillus*, *Bifidobacterium*, and *Saccharomyces*. These microorganisms offer several health benefits^[11,12]^, including (i) antimicrobial effects^[13]^; (ii) anti-inflammatory effects^[14,15]^; (iii) antioxidant properties^[16]^; (iv) immunomodulation activities^[11]^; (v) treatment of diarrhea^[11]^; (vi) fighting antimicrobial resistance^[17]^; (vii) prevention of heavy metal toxicity^[18]^; and (viii) reduction of colon cancer risk^[19]^. Indeed, probiotic bacteria survive the harsh conditions of the GIT, and after oral administration, these exogenous bacteria may reach numbers similar to those of gut commensals^[20]^. Such transitory or “visiting” microorganisms do not colonize the host^[21]^, but when ingested in sufficient amounts, they modulate the gut microbiota and influence human health. Recently, next-generation probiotics have been described as specific commensal bacterial strains found in the GIT, such as *Bacteroides*, *Clostridium*, *Faecalibacterium*, and *Akkermansia*^[22,23]^.

**Figure 1:**
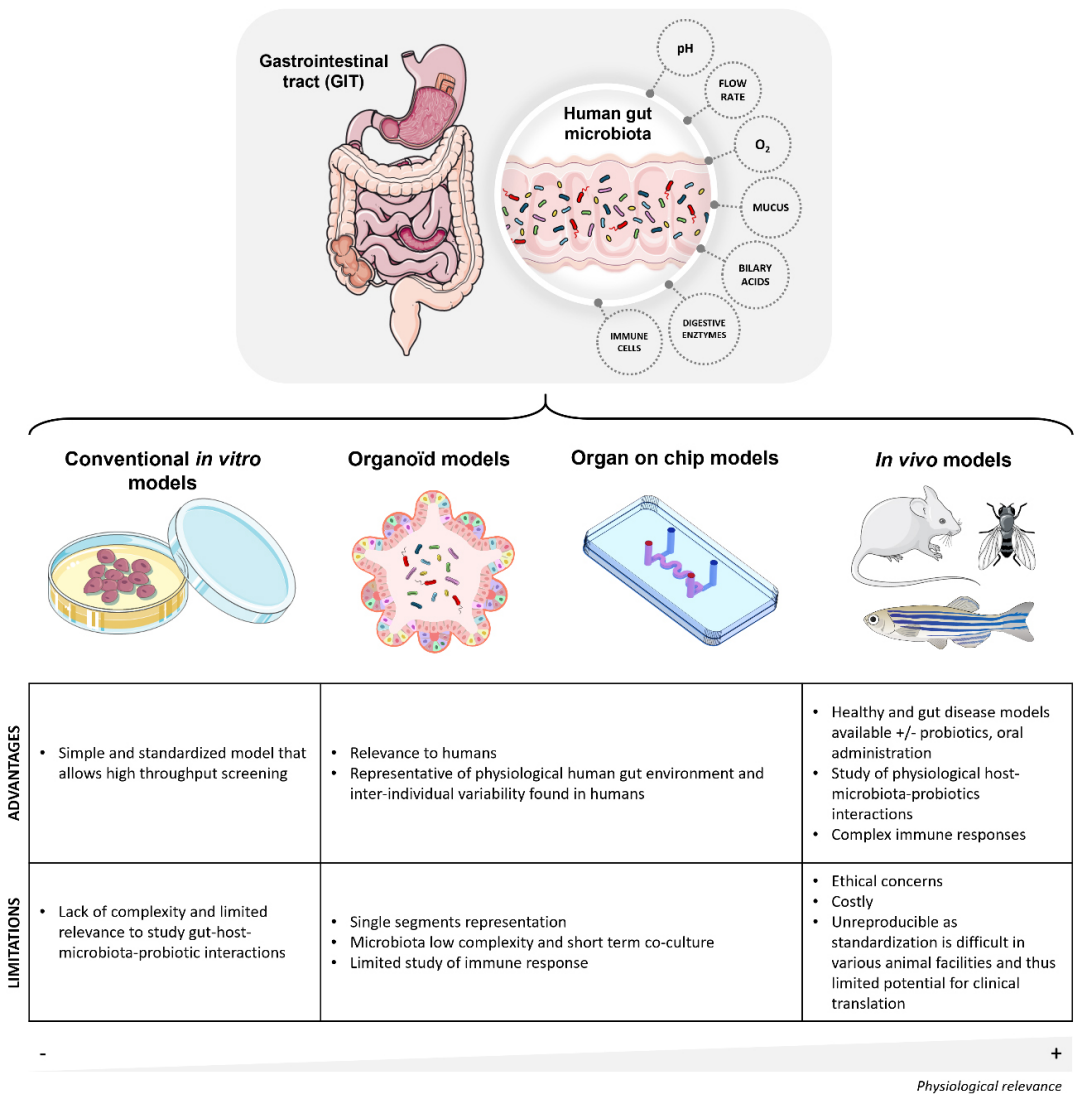
Schematic illustration of the human gastrointestinal tract and overview of the major gut models used to study host interactions between the host, the microbiota, and probiotics. The gastrointestinal tract comprises different compartments where probiotics and gut microbiota encounter various challenges. Gut models include conventional *in vitro* models, organoid models, organ-on-chip models and *in vivo* models. The annotations provide insights into the advantages and the limitations of each model. The above figure was modified from the Servier Medical Art (https://smart.servier.com/), licensed under CC BY 4.0.

Significant knowledge has been gained regarding the complexity of the intestinal microbiota and probiotics during the past two decades, thanks to the progress of multi-OMICS and bioinformatics approaches, including metagenomics, proteomics, and metabolomics^[24-26]^. These approaches have uncovered the specific identity and quantity of gut microbes in human specimens. The main challenge is now to translate these findings into health and disease contexts and to move from “association and correlation” to “causality” assessments. Until now, animal models have been the cornerstone for interrogating and gaining mechanistic insights into the drivers and regulators of intestinal homeostasis^[27]^. Interestingly, *in vitro* models, including organoids and organ-on-chip (OOC) systems, have advanced substantially over the last decade, offering new perspectives for precision health modeling, including the study of the microbiota. In this article, we discuss how gut models, ranging from animal models to gut-on-chip (GOC) technologies, can advance our understanding of the functional analysis of microbiota and diversity [Figure 1].

## FROM ANIMAL MODELS TO ORGAN-ON-CHIP TECHNOLOGY

### Mouse, a cornerstone model for studying gut interactions between the host, the microbiota and orally administered probiotics

Germ-free mice colonized with engineered bacterial communities, also known as defined or synthetic microbiota, are invaluable models for studying host-microbiota interactions under controlled conditions^[28,29]^. However, the standardization of microbiota transfer poses challenges, including variability in microbial composition among mice, which impacts reproducibility. Selective colonization of germ-free mice with defined complex human bacterial communities is one possible way to overcome these limitations^[30]^. Similarly, mice have been instrumental in investigating host-probiotic interactions and the potential translation to human clinical trials^[31]^. Indeed, the gut microbiota of humans and rodents is similarly dominated by *Firmicutes* (currently *Bacillota*) and *Bacteroidota* (currently *Bacteroidetes*) phyla, followed by *Actinobacteria* (currently *Actinomycetota)* and *Proteobacteria* (currently *Pseudomonadota)*. Moreover, mice are very good models for the use of small animal imaging techniques to monitor microbial behavior in real time^[32,33]^. However, (i) ethical considerations; (ii) high costs; and (iii) divergence among laboratory mouse models from different suppliers and research institutions may lead to a lack of reproducibility and conflicting data in basic and preclinical research. These factors can also limit their applicability.

### Non-rodent animal models for studying gut interactions between the host, the microbiota and administered probiotics

Non-rodent animal models have also been developed for investigating microbiota-host interactions. The zebrafish (*Danio rerio*) is attracting interest because its intestinal structure and function are highly similar to those of mammals^[34]^. Its low cost, the possibility of using germ-free larvae derived from non-axenic parents, and the capacity to study transparent larvae and adults from different genetic lines with imaging technology make this model highly valuable. Recently, it was suggested that the stage of development strongly influences gut microbial composition^[35]^. Moreover, probiotic administration modulates the gut microbial community. The fruit fly *Drosophila melanogaster* is also now increasingly used to study interactions between the host, the gut microbiota, and orally administered probiotics^[36,37]^. Its microbiota is mainly composed of culturable aerotolerant bacteria, yeasts and fungi. Due to its simplicity, short lifecycle, and well-characterized genetics, the fruit fly serves as a high-throughput model for studying probiotics, microbiota and host interactions.

### Current limitations and future perspectives of animal models for studying probiotics, microbiota and the host

Monitoring microbiota and probiotic strains within the complex environments of animal models requires new advanced fluorescent protein labeling and high-resolution imaging techniques^[38-41]^. Moreover, investigating host-microbe interactions is challenging as the relationship between a given member of the microbiota and the host is influenced by different parameters such as genetics and immune system reactivity^[42]^. Thus, these models are limited in their ability to reflect the inter-individual variability observed in the human gut environment^[43]^.

### The emergence of organ-on-chip technologies

Recently, OOC technologies have emerged as a promising alternative to animal experiments^[44]^. Unlike traditional two-dimensional (2D) cell culture models, these microphysiological systems closely mimic human physiology, including immune responses and infection dynamics^[45,46]^. In contrast to animal models, which often show significant differences from human physiology, OOCs help overcome these limitations and provide new opportunities to explore complex host-microbe interactions. For instance, by replicating key physiological features of the intestine, such as flow rate, oxygen gradients, and peristaltic mechanical forces, various GOC technologies have enabled the study of complex bacterial communities that conventional static *in vitro* cell culture models fail to reproduce^[47,48]^. In today’s landscape, numerous GOC models have been developed. While GOC models may employ different approaches, they all share the common feature of controlling the intestinal microenvironment within a microfluidic chip. One of the first models developed by Ingber’s lab was based on a polydimethylsiloxane (PDMS) chip with two culture chambers separated by a porous flexible membrane^[49,50]^. The upper chamber cultures of intestinal cells are under continuous nutrient flow, while the lower chamber cultures mimic an underlying endothelium or stromal compartment. The originality of this model also includes the capacity to recapitulate peristaltic motions using two lateral chambers. Application of physiological vacuum stretches the flexible supportive membrane, thereby simulating peristalsis. Additionally, the exposure of physiological flow in the system induces self-organization of intestinal cells into three-dimensional structures resembling intestinal villi and crypts. While PDMS is well characterized and cost-effective, this material resists changes in shape and is prone to adsorbing small hydrophobic molecules. Some other groups have also developed other GOC models to increase the biocompatibility and physiological relevance. For instance, the topology of the intestinal structures can be further refined using specialized microfabricated molds that closely reconstitute villi and crypts^[51-54]^. Using hydrogels, this approach also allows tailoring the substrate to closely match the stiffness of the intestinal stroma, thereby optimizing the cellular microenvironment. While these new models are more challenging to produce, their higher physiological fidelity may offer advantages for studying host-microbe interactions.

### Gut-on-chip for studying probiotics, microbiota and the host

**Figure 2:**
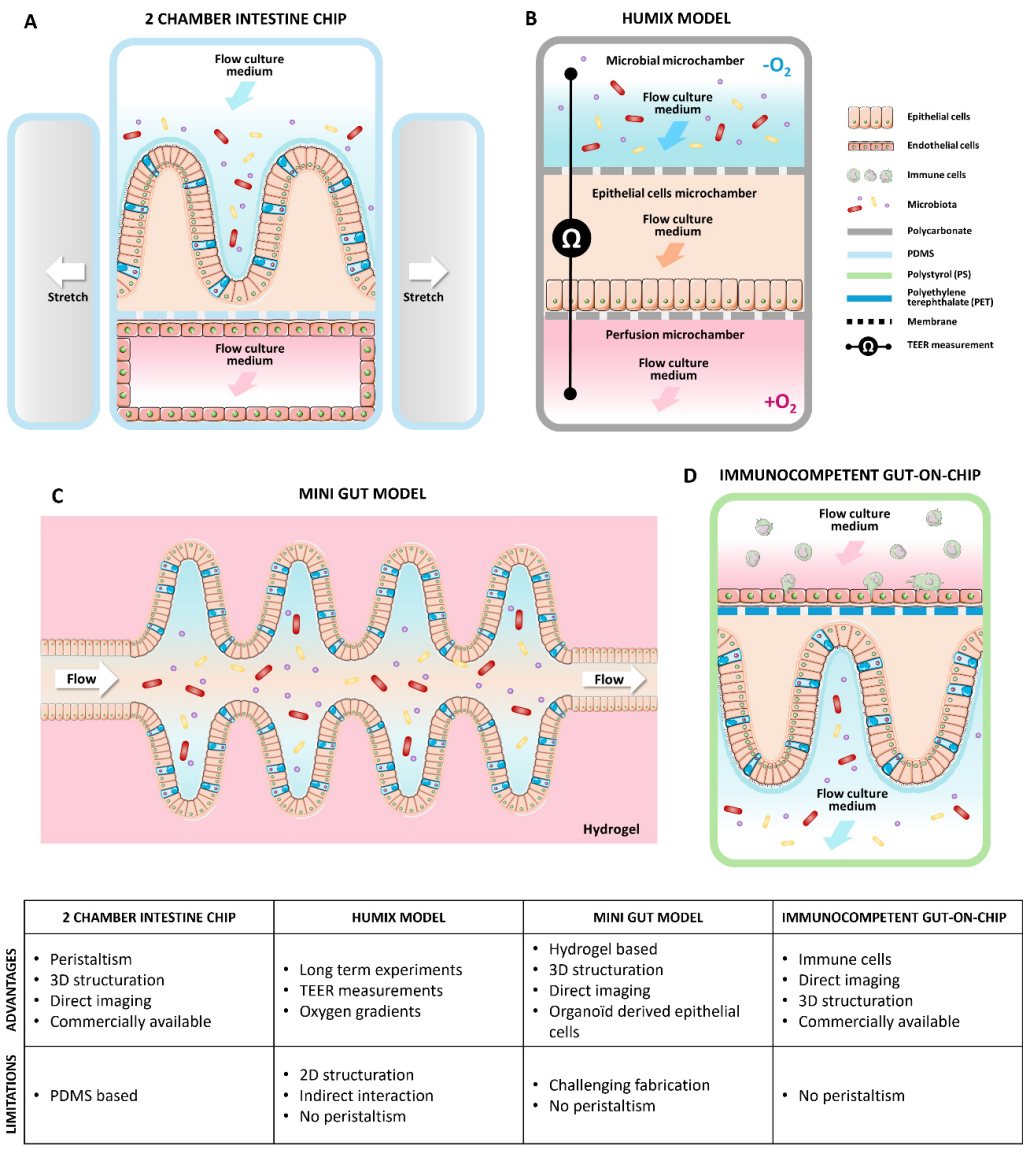
GOC models used to study gut interactions between the host, the microbiota, and orally administered probiotics *in vitro*, along with their advantages and limitations. (A) Commensal Intestine chip with peristaltism from Emulate Company^[50]^; (B) The HuMix gut-on-chip model with three superposed culture microchambers and integrated sensors^[63]^; (C) Mini gut fabricated by laser ablation of a 3D hydrogel^[52]^;(D) Commercial immunocompetent 2 channels gut-on-chip model^[70]^. The above figure was modified from the Servier Medical Art (https://smart.servier.com/), licensed under CC BY 4.0. GOC: Gut-on-chip.

Using the pioneering model based on two-cell culture stacked channels^[50]^, host-microbiota interactions have been investigated by introducing bacterial species directly into the intestinal lumen (upper channel). The feasibility of co-culturing mono-associations such as *Bacteroides fragilis* or *Escherichia coli*^[55-57]^ and a defined probiotic consortium (e.g., VSL#3 mixture) has been demonstrated^[50]^. Future improvements to GOC devices, including the integration of oxygen gradients, will enable co-cultivation of more complex human fecal microbiota in an anaerobic microenvironment^[58,59]^. The integration of hypoxia and a mucus layer allowed the sustained growth of obligate anaerobic bacteria, such as *B. fragilis,* for up to 3 days without major alterations in intestinal barrier integrity. Furthermore, 16S rRNA sequencing analysis identified the diversity and relative abundance of the microbiota co-culture in GOC. With over 200 unique Operational Taxonomic Units (OTUs) identified, this study opened new perspectives for investigating complex microbiota co-cultures in the GOC model^[59]^. While not fully explored yet, GOC also offers a new visual window to monitor the spatio-temporal dynamics of this ecosystem^[60]^. Recently, a new GOC model has been optimized for high-resolution imaging, enabling long-term imaging for visualizing *Lactiplantibacillus plantarum* colonization of the epithelium interface^[61]^. Several other GOC technologies have also been developed for studying the impact of microbiota^[62,63]^. For instance, the HUMIX system, developed by Paul Wilmes and colleagues, consists of a three-chamber fluidic culture system in which a semipermeable membrane separates a microbial chamber from an epithelial chamber^[63]^. The third and final chamber serves for nutrient exchange. This design allows for metabolite exchange and oxygen control but does not simulate mechanical peristalsis. Since the microbiota is not in direct contact with the epithelium, this approach enables the exploration of the impact of microbial metabolites and other secreted products. Importantly, most studies so far have been based on Caco-2 cell lines, as traditionally employed in 2D cultures. However, recent advances have shown that using patient-derived organoids as a primary cell source can significantly enhance physiological relevance^[64,65]^. Similarly, few studies have used human-induced pluripotent stem cells to generate more realistic intestinal tissues^[66]^. Future integration of organoids with OOC models could help to further understand the impact of microbiota and probiotics on the intestinal tract. Alternatively, another microphysiological system integrating a human explant, called the Intestinal Explant Barrier Chip, has been developed^[67]^. Using this platform, the same group has demonstrated the beneficial effects of gut microbiota following inulin treatment^[68]^. Overall, the diversity of GOC models [Figure 2] now provides new opportunities to explore the dynamics of host-microbiota interactions under defined and physiological conditions in humans.

### Limitations and future perspectives of organ-on-chip for studying microbiota

Despite rapid advances in GOC models, several limitations remain. First, most systems are based on PDMS, a biocompatible, low-cost material that facilitates prototyping and academic use. However, its tendency to adsorb small hydrophobic molecules limits its suitability for pharmaceutical applications. Therefore, the development of more predictive and biocompatible materials is highly desirable. Recent advances in hydrogel-based chips provide a promising alternative in the field of OOC^[52]^. Using an in-house designed hydrogel, the capacity to grow stem cell-based GOC has also been demonstrated. Second, the gut is compartmentalized into different sections, from the stomach to the colon. Unlike *in vivo* models or advanced *in vitro* models such as Simulator of the Human Intestinal Microbial Ecosystem (SHIME®)^[69]^, no current OOC fully replicates the entire digestive tract. The SHIME® model is a multi-compartment simulator that replicates the human GIT, including the stomach, small intestine, and the colon^[69]^. It maintains controlled conditions of pH, temperature, and anaerobiosis, enabling the cultivation of a complex and stable microbial community. As different individual OOC models mimicking the stomach, duodenum, jejunum and colon have been developed, integrating a miniaturized SHIME® system on a chip would be an interesting technological development. Organoid technologies combined with microfluidic dynamics offer promising avenues for developing multi-OOC systems that mimic the entire GIT. Third, immune system integration remains a major challenge for OOC platforms. Recent strategies have included perfusing patient-derived peripheral blood mononuclear cells into endothelial channels or adding pro- or anti-inflammatory cytokines^[62,70]^. New approaches, such as autologous immune cell integration, could further enhance our understanding of the necessary tripartite interactions between the microbiota, epithelium, and immune system.

Finally, the rise of artificial intelligence and *in silico* modeling will help to refine and optimize preclinical models using both animals and organ(oid)-on-chip systems^[71]^. The standardization of protocols, the study of synergies, and the integration of multiple complementary models will help compensate for the limitations of each individual model^[72]^ [Figure 2]. This will then lead to improved modeling of human conditions and greater reproducibility, facilitating personalized microbiota and probiotic therapies.

## DECLARATIONS

### Copyright

© The Author(s) 2025.
